# The Role of Circulating Collagen Turnover Biomarkers and Late Gadolinium Enhancement in Patients with Non-Ischemic Dilated Cardiomyopathy

**DOI:** 10.3390/diagnostics12061435

**Published:** 2022-06-10

**Authors:** Radu Revnic, Bianca Olivia Cojan-Minzat, Alexandru Zlibut, Rares-Ilie Orzan, Renata Agoston, Ioana Danuta Muresan, Dalma Horvat, Carmen Cionca, Bogdan Chis, Lucia Agoston-Coldea

**Affiliations:** 1 Department of Family Medicine, Iuliu Hatieganu University of Medicine and Pharmacy, 400012 Cluj-Napoca, Romania; radu_revnic@yahoo.com (R.R.); cojanminzat.bianca@yahoo.com (B.O.C.-M.); 2 Department of Internal Medicine, Iuliu Hatieganu University of Medicine and Pharmacy, 400012 Cluj-Napoca, Romania; orzanrares@gmail.com (R.-I.O.); ioanamuresandanuta@yahoo.com (I.D.M.); hdalma92@yahoo.com (D.H.); bogdan_a_chis@yahoo.com (B.C.); luciacoldea@yahoo.com (L.A.-C.); 3 Faculty of Medicine, Iuliu Hatieganu University of Medicine and Pharmacy, 400012 Cluj-Napoca, Romania; renata.agoston@yahoo.com; 4 Department of Radiology, Affidea Hiperdia Diagnostic Imaging Centre, 400487 Cluj-Napoca, Romania; carmen.cionca@gmail.com; 5 Department of Internal Medicine, Emergency County Hospital, 400347 Cluj-Napoca, Romania

**Keywords:** cardiac magnetic resonance imaging, galectin-3, procollagen type I carboxy-terminal pro-peptide, N-terminal pro-peptide of procollagen type III

## Abstract

Background: Myocardial scarring is a primary pathogenetic process in nonischemic dilated cardiomyopathy (NIDCM) that is responsible for progressive cardiac remodeling and heart failure, severely impacting the survival of these patients. Although several collagen turnover biomarkers have been associated with myocardial fibrosis, their clinical utility is still limited. Late gadolinium enhancement (LGE) determined by cardiac magnetic resonance imaging (CMR) has become a feasible method to detect myocardial replacement fibrosis. We sought to evaluate the association between collagen turnover biomarkers and replacement myocardial scarring by CMR and, also, to test their ability to predict outcome in conjunction with LGE in patients with NIDCM. Method: We conducted a prospective study on 194 patients (48.7 ± 14.3 years of age; 74% male gender) with NIDCM. The inclusion criteria were similar to those for the definition of NIDCM, performed exclusively by CMR: (1) LV dilation with an LV end-diastolic volume (LVEDV) of over 97 mL/m^2^; (2) global LV dysfunction, expressed as a decreased LVEF of under 45%. CMR was used to determine the presence and extent of LGE. Several collagen turnover biomarkers were determined at diagnosis, comprising galectin-3 (Gal3), procollagen type I carboxy-terminal pro-peptide (PICP) and N-terminal pro-peptide of procollagen type III (PIIINP). A composite outcome (all-cause mortality, ventricular tachyarrhythmias, heart failure hospitalization) was ascertained over a median of 26 months. Results: Gal3, PICP and PIIINP were considerably increased in those with LGE+ (*p* < 0.001), also being directly correlated with LGE mass (r^2^ = 0.42; r^2^ = 0.44; r^2^ = 0.31; all *p* < 0.001). Receiver operating characteristic (ROC) analysis revealed a significant ability to diagnose LGE, with an area under the ROC of 0.816 for Gal3, 0.705 for PICP, and 0.757 for PIIINP (all *p* < 0.0001). Kaplan–Meier analysis showed that at a threshold of >13.8 ng/dL for Gal3 and >97 ng/dL for PICP, they were able to significantly predict outcome (HR = 2.66, *p* < 0.001; HR = 1.93, *p* < 0.002). Of all patients, 17% (*n* = 33) reached the outcome. In multivariate analysis, after adjustment for covariates, only LGE+ and Gal3+ remained independent predictors for outcome (*p* = 0.008; *p* = 0.04). Nonetheless, collagen turnover biomarkers were closely related to HF severity, providing incremental predictive value for severely decreased LVEF of under 30% in patients with NIDCM, beyond that with LGE alone. Conclusions: In patients with NIDCM, circulating collagen turnover biomarkers such as Gal3, PICP and PIIINP are closely related to the presence and extent of LGE and can significantly predict cardiovascular outcome. The joint use of LGE with Gal3 and PICP significantly improved outcome prediction.

## 1. Introduction

Despite recent therapeutic developments, non-ischemic dilated cardiomyopathy (NIDCM) remains a primary cause of progressive cardiac remodeling and heart failure (HF) that leads to frequent hospitalization and increased mortality. These patients have an increased risk of developing myocardial fibrosis, which, in turn, plays a central role in the progression of HF [1]. Cardiac magnetic resonance imaging (CMR) with late gadolinium enhancement (LGE) detects focal replacement myocardial fibrosis in up to 30% of patients with NIDCM and provides an incremental predictive value for cardiovascular risk stratification [2]. However, using this technique, diffuse interstitial fibrosis remains undetected [3].

Molecular markers of fibrosis such as galectin-3 (Gal3), procollagen type I carboxy-terminal pro-peptide (PICP) and N-terminal pro-peptide of procollagen type III (PIIINP) are in direct relationship with myocardial collagen turnover, and thus might aid in the prediction of major adverse cardiovascular events (MACEs) [4]. Gal3 binds to a specific beta-galactosidase which is overexpressed by phagocytic macrophages, and thus endorses the proliferation of myofibroblasts with myocardial collagen deposition—leading to the progression of myocardial fibrosis, inflammation, fibrosis and cardiac remodeling [5,6,7,8,9,10]. Moreover, increased serum levels of Gal3 have been identified in patients with chronic HF [5,11,12,13] and also predicts cardiac remodeling and mortality in this category of patients [7,8,14]. Additionally, in one recently published study, it has been shown that sera levels of Gal3 were closely associated with the extent of LGE in patients with NIDCM [15].

To date, PICP and PIIINP are the only proven peptides that can be identified in the bloodstream that are considerably correlated to histologically proven myocardial fibrosis [4]. These molecules have been observed to be considerably correlated to the progression of myocardial fibrosis in patients with ischemic heart disease and NIDCM [7,8]. Additionally, their increased sera levels are able to predict MACEs in patients with HF and preserved left ventricle (LV) ejection fractions (LVEFs) [4,16]. However, their role in patients with NIDCM is not entirely clear.

The aim of this study was to evaluate the link between circulating collagen turnover biomarkers and myocardial replacement fibrosis determined by CMR, and also to test their ability to predict outcome in conjunction with LGE.

## 2. Materials and Methods

### 2.1. Study Design and Patient Characteristics

**Figure 1:**
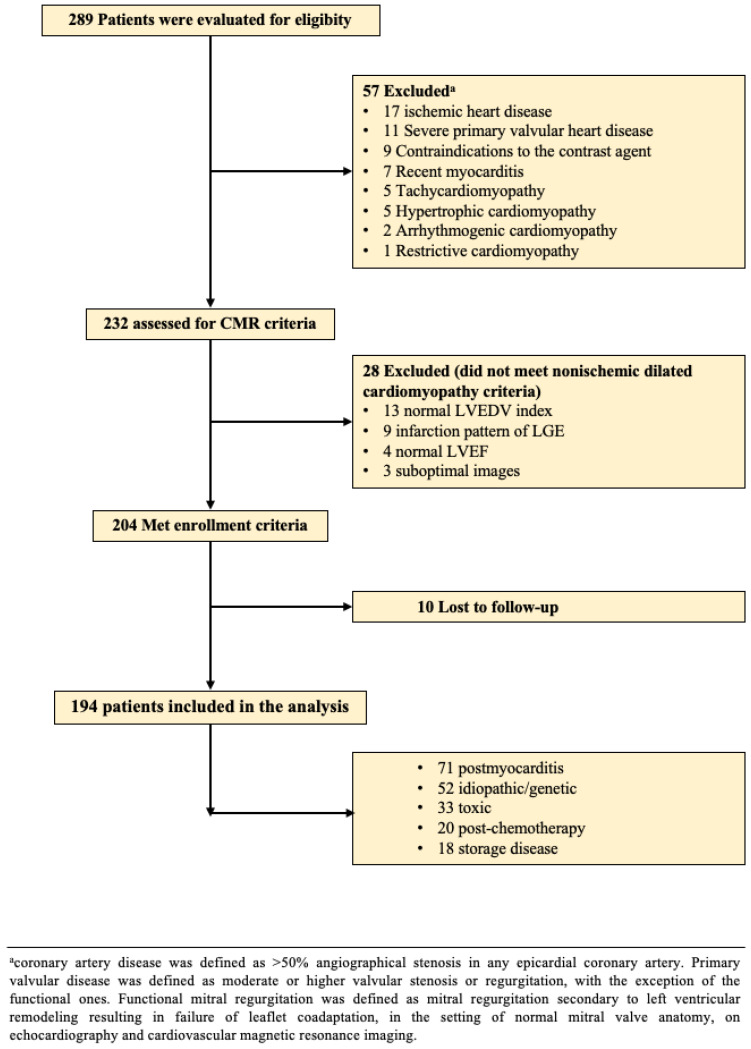
Flowchart of the study.

We conducted an observational, prospective study on patients recently diagnosed with NIDCM who were examined in the Department of Internal Medicine, Iuliu Hatieganu University of Medicine and Pharmacy of Cluj-Napoca, between October 2017 and November 2020. The inclusion criteria were similar to those for the definition of NIDCM, performed exclusively by CMR: (1) LV dilation with an LV end-diastolic volume (LVEDV) of over 97 mL/m^2^; (2) global LV dysfunction, expressed as a decreased LVEF of under 45% [17]. The exclusion criteria are presented in Figure 1. The current study was conducted in accordance with the Declaration of Helsinki and received approval from the Ethics Committee of Iuliu Hatieganu University of Medicine and Pharmacy of Cluj-Napoca, Romania. All patients were fully informed about the study protocol and provided written consent.

All patients underwent a similar investigation protocol, which included demographic and clinical data and biological sampling, along with standard cardiovascular evaluation and CMR.

### 2.2. Circulating Collagen Turnover Biomarkers

Biochemical workups were performed in the Clinical Biochemistry Laboratory from the 2nd Medical Clinic of the Cluj Emergency County Hospital. Two peripheral venous blood samples were harvested, centrifuged immediately after, and stored in special vials with ethylenediaminetetraacetic acid. Sera glucose and creatinine were determined from one vial using a Konelab-31 analyzer, while the other vial was stored at −70 °C until the end of the study and used to determine the serum levels of cardiac biomarkers. PICP, PIIINP, copeptin (CPP) and N-terminal pro-Brain Natriuretic Peptide (NT-proBNP) were determined by the Sandwich ELISA technique according to the manufacturer’s recommendations using Elabscience Biotechnology Co., Ltd, Wuhan, China. The inferior thresholds and variabilities for these markers were as follows: for PICP, a cut-off value of 0.13 ng/mL and inter-/intra-test variability <10%/<12%; for PIIINP, a cut-off value of 0.14 ng/mL and inter-/intra-test variability <10%; for CPP, a cut-off value of 0.18 ng/mL and inter-/intra-test variability <10%; for NT-proBNP, a cut-off value of 0.38 ng/mL and inter-/intra-test variability <10%. Serum levels of Gal3 were measured using an enzyme-linked immunosorbent assay (Human Galectin-3—Quantikine ELISA Kit, R&D Systems), with an inferior cut-off value of 0.016 ng/mL, without crosslinked reactivity with other galectin or collagen molecules. Intra-test and inter-test plasma variations for Gal3 were 3.5–4.3%, and 5.8–6%, respectively. Renal function was evaluated using the estimated glomerular filtration rate (eGFR) and renal impairment was considered as an eGFR of under 60 mL/min/1.73 m^2^.

### 2.3. CMR Measurements

CMR images were appraised using a 1.5 T Open Bore system MR scanner (Magnetom Altea, Siemens Medical Solutions, Erlangen, Germany) in complete apnea by two level-III experienced operators who were blinded to all clinical and imaging data, in line with current international guidelines [18]. The acquisition of steady-state free precession (SSFP) CMR sequences was performed to detect ventricular function and mass using standard long- and short-axes (two-chamber, three-chamber, and four-chamber) to enclose both ventricles were covered from the base to the apex. Cine-SSFP parameters were as follows: repetition time (TR) 3.6 ms; echo time (TE) 1.8 ms; flip angle 60°; slice thickness 6 mm; field-of-view 360 mm; image matrix of 192 × 192 pixels; voxel size 1.9 × 1.9 × 6 mm; 25–40 ms temporal resolution reconstructed to 25 cardiac phases.

Focal myocardial fibrosis was evaluated by LGE detected at 10 min after intravenous infusion of 0.2 mmol/kg gadoxetic acid (Clariscan, GH Healthcare AS, Oslo, Norway) using long- and short axis-views, using a segmented inversion-recovery gradient-echo sequence. LGE acquisition parameters were TR 4.8 ms, TE 1.3 ms and inversion time 200 to 300 ms. Inversion time was adjusted to optimize nulling of normal myocardium. Brachial blood pressure was monitored during SSFP-CMR acquisitions.

LVEDV, LV end-systolic volume (LVESV), LVEF and end-diastolic LV mass (LVM) were measured on short-axis cine-SSFP images. Epicardial and endocardial borders were traced semi-automatically at end-diastole and end-systole using a Syngo Virtual Cockpit. All volumes were indexed to body surface area (BSA). Besides this, for a more accurate assessment of LV function, we assessed the LV longitudinal-axis strain (LV-LAS; the difference in mitral annular displacement at end-systole vs. end-diastole expressed as a percentage) and LV sphericity index (LVSI). The LVSI was calculated by dividing LVEDV by the volume of a sphere, whose LV length (L) was measured at the end-diastole: LVSI = LVEDV/(π/6 × (L)^3^) [19,20].

The presence and distribution of LGE in the LV were assessed from short-axis images using the 17-segments model, as recommended by the American Heart Association [21], and quantified using a signal intensity threshold of >5 standard deviations (SDs) above a remote reference of the normal myocardium. This threshold proved to be in the best agreement with visual assessments and had the best reproducibility among the different technique thresholds [22]. The Full Width at Half Maximum (FWHM) technique was used to quantify LGE. The reference region was defined as an area that included the maximum intensity of the visually appreciated LGE signal on each slice. The maximum signal strength threshold was recorded to define LGE. Additionally, the total LGE was determined as the sum of all LGE areas for each slice, multiplied by the slice thickness. LGE quantification was performed by 2 independent observers. Inter-observer reproducibility was 0.91 95% CI (0.882–0.934) and intra-observer reproducibility was 0.93 95% CI (0.902–0.947). Specific LGE distribution patterns were accounted for: mid-wall or subepicardial, and focal or diffuse. The LGE mass was automatically quantified from short-axis LGE images, using cvi42, Circle Cardiovascular Imaging Inc., Calgary, CA, Canada. The extent of LGE was expressed in grams (g) and percentage of LV mass.

### 2.4. Clinical Outcome

The clinical follow-up was obtained by completing a questionnaire either during hospital visits, telephone house-calls, or both—aiming to delineate the occurrence of clinical outcomes, which corresponded to the first event occurring in each patient among the following MACEs: death or aborted death from cardiac causes, sustained ventricular tachyarrhythmia (beats with ventricular origin that last >30 s and have a rate greater than >100 beats/min), and HF requiring hospitalization—defined according to current international guidelines. Hospitalization due to non-cardiac causes was not counted as an event. Survival analysis was performed for the clinical outcomes. The median follow-up was 26 months and maximum follow-up reached 41 months.

### 2.5. Statistical Analysis

Initially, the Kolmogorov–Smirnov test was used to assess data normality. Continuous data were presented as median (inter-quartile range (IQR)) and mean ± standard deviation (SD). Discrete data were reported as percentages and frequencies. The distribution of variables was accounted for after logarithmic transformation. Comparisons between groups were approached using Hi^2^ and Fischer tests for qualitative data and ANOVA or Kruskal–Wallis H tests for continuous data. Pearson’s coefficient of correlation was used to examine the relationship between data. Furthermore, for specific descriptive analyses, the studied population was dichotomized according to LGE presence (LGE+) or absence (LGE−), and also with respect to higher than median levels of PICP, PIIINP and Gal3 (PICP+, PIIINP+, Gal3+) and lower than median levels of these biomarkers (PICP−, PIIINP−, Gal3−)—thus resulting in six specific groups. Also, logistic regression was used to evaluate the incremental ability of these markers.

Kaplan–Meier survival curves were created and differences between groups were assessed using log-rank tests. Unadjusted and adjusted Cox regression analysis was performed to determine hazard rates (HRs) and 95% confidence intervals (CIs). Furthermore, adjustment regression models were used to test if the biomarkers of cardiac fibrosis did or did not respect a linear trend. For the adjustment, specific covariates which are known to significantly predict outcome in patients with NIDCM such as LVEF, eGFR, body-mass index (BMI), NT-proBNP, diabetes, gender and age were used. Moreover, ROC analyses were used to calculate the cut-off values of circulating biomarkers for predicting MACEs.

Additionally, inter- and intra-observer Kappa Cohen coefficients were calculated. Retrospective calculus of statistical test powers and prospective dimensions of the sample were estimated using type I and type II variations, based on sample size. The statistical analysis was performed using statistical software MedCalc (Version 19.1.7, MedCalc Software, Ostend, Belgium).

## 3. Results

### 3.1. Baseline Characteristics

A total of 194 patients with NIDCM were enrolled in the study and their main characteristics are presented in Table 1. They were divided into two groups based on the presence and absence of LGE: 73 (37.7%) patients were LGE+ and 121 (62.3%) were LGE−. Those in the LGE+ group had significantly increased sera levels of circulating collagen turnover biomarkers—namely Gal3, PICP and PIIINP—compared to the others: 17.7 ng/mL vs. 9.1 ng/mL, *p* < 0.001; 156 ng/mL vs. 74 ng/mL, *p* < 0.001; and 5.1 ng/mL vs. 3.5 ng/mL, *p* < 0.001, respectively. Moreover, patients with LGE+ had significantly modified LVEDV (142.4 mL/m^2^ vs. 124.2 mL/m^2^, *p* < 0.001), LVESV (101.2 mL/m^2^ vs. 78.1 mL/m^2^, *p* < 0.001), LVSI (0.44 vs. 0.38, *p* < 0.001) and LV-LAS (−8.5 vs. −10.5%, *p* < 0.001), and significantly lower LVEF (30.3% vs. 38.2%, *p* < 0.001).

### 3.2. Association between Circulating Collagen Turnover Biomarkers and LGE

**Figure 2:**
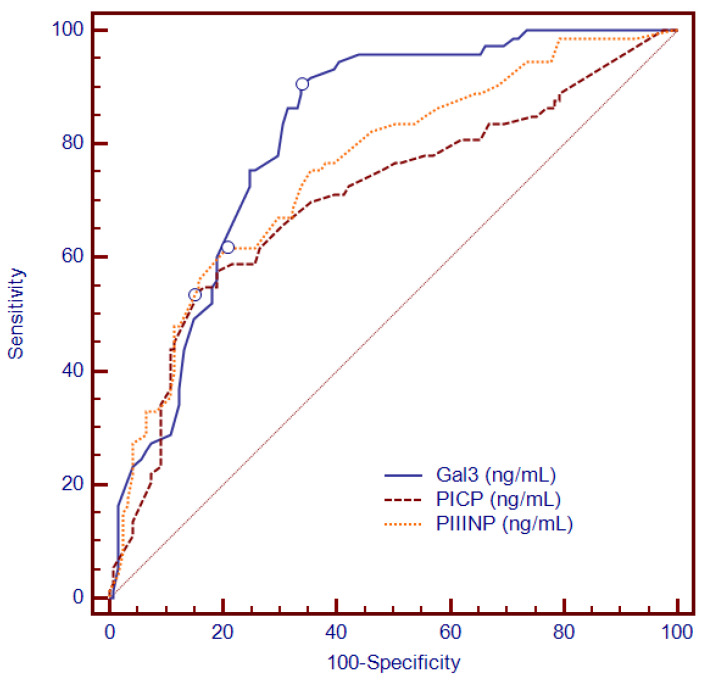
ROC analysis demonstrating the ability of Gal-3, PICP and PIIINP to identify the presence of LGE. Abbreviations: Gal-3, galectin-3; LGE, late gadolinium enhancement; PICP, procollagen type I carboxy-terminal pro-peptide; PIIINP, N-terminal pro-peptide of procollagen type III; ROC, receiver operating characteristics.

Overall, sera levels of PICP, PIIINP and Gal-3 were significantly increased in patients with LGE (LGE+), as compared to those without LGE (Table 1). Furthermore, Gal3, PICP and PIIINP were positively associated with LGE mass, with significant Pearson’s correlation coefficients of r^2^ = 0.372, *p* < 0.0001; r^2^ = 0.379, *p* < 0.0001; and r^2^ = 0.315, *p* < 0.0001. ROC analysis demonstrated that specific cut-off values significantly identified the presence of LGE: an area under the ROC of 0.816 for Gal3 (95% CI: 0.754–0.868; *p* < 0.0001), 0.705 for PICP (95% CI: 0.636–0.769; *p* < 0.0001) and of 0.757 for PIIINP (95% CI: 0.690–0.816, *p* < 0.0001; Figure 2). Additionally, an 11 ng/mL threshold for Gal3 revealed the presence of LGE with a high sensitivity of 90.4%, a specificity of 66.1% and a negative predictive value of 92%, while PIIINP proved—for a cut-off value of 1.18 ng/mL—to have a sensitivity of 71.83%, a specificity of 83% and a negative predictive value of 83.6%, and PICP had—for a cut-off value of 44.4 ng/mL—a 77.5% sensitivity, 76% specificity and a negative predictive value of 85.5%.

Regarding the relationship between circulating collagen turnover biomarkers and the severity of HF, Gal3 and PICP were inversely correlated with LVEF (r^2^ = −0.58, *p* < 0.0001; r^2^ = −0.39, *p* < 0.0001) and NYHA class ≥III (r^2^ = −0.56, *p* < 0.0001; r^2^ = −0.39, *p* < 0.0001), and directly correlated with NT-proBNP (r^2^ = 0.49; *p* < 0.0001; r^2^ = 0.32; *p* < 0.0001) and CCP (r^2^ = 0.41; *p* < 0.0001; r^2^ = 0.38; *p* < 0.0001) levels.

### 3.3. Characterization of Patients with DCM and Severely Decreased LV Function

As shown in Table 2, 31% (*n* = 61) of patients had a severely decreased LVEF of under 30%. These patients had significantly impaired LVEDV, LVESV, LVSI and LV-LAS (all *p* < 0.001) and considerably higher LGE mass (31.2 vs. 6.4, *p* < 0.001) and LGE mass/LV mass ratios (18.2 vs. 4.5, *p* < 0.001), as compared to those with LVEFs over 30%.

Sera markers of HF were notably increased in those with LVEF < 30%: NT-proBNP: 17,500 ng/L vs. 16,200 ng/L, *p* < 0.001 and CPP: 17.5 ng/mL vs. 9.5 ng/mL, *p* < 0.001, respectively.

**Figure 3:**
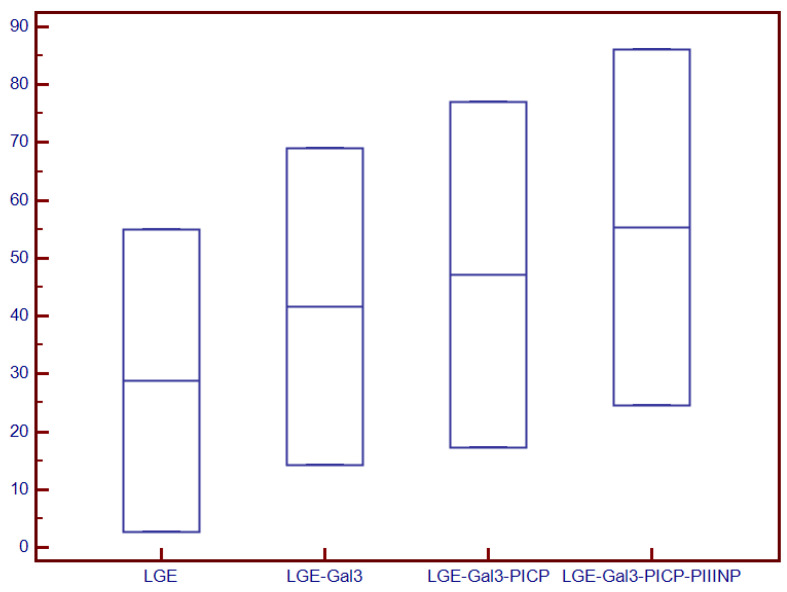
Incremental ability of LGE, LGE stepwise added to Gal-3, PICP and PIIINP for identifying patients with NIDCM and severely decreased LVEF. Abbreviations: Gal-3, galectin-3; LGE, late gadolinium enhancement; PICP, procollagen type I carboxy-terminal pro-peptide; PIIINP, N-terminal pro-peptide of procollagen type III; ROC, receiver operating characteristics.

A stepwise logistic regression proportional-hazard model analysis was deployed to test if collagen turnover biomarkers are useful in the risk stratification of patients with NIDCM and severely decreased LVEF, beyond LGE. LGE alone significantly predicted the presence of LVEF <30% in patients with NIDCM (Chi-square = 55.72, *p* < 0.0001). The addition of Gal3 to LGE significantly increased the diagnosis power (Chi-square = 69.69, *p* < 0.0001), while further adding PICP increased their identification ability even more (Chi-square = 79.31, *p* < 0.0001). Lastly, the association of Gal3, PICP and PIIINP with LGE provided a significant incremental value for predicting decreased LVEFs of <30% in patients with NIDCM (Chi-square = 86.09, *p* < 0.0001; Figure 3).

### 3.4. Univariate and Multivariate Cox Analysis and Time-To-Event Analysis of LGE and Circulating Collagen Turnover Biomarkers

Patients were followed up for 26 months. Of them, 17% (*n* = 33) of patients reached the outcome: all-cause mortality (*n* = 6), malignant ventricular tachyarrhythmia (*n* = 14) and HF hospitalization (*n* = 13; Table 3).

In the univariate Cox analysis, LGE and all cardiac biomarkers of fibrosis (Gal3, PICP and PIIINP) were significantly associated with MACEs. However, following the multivariate analysis, after adjustment for covariates comprised of age, gender, LVEF, eGFR, BMI, NT-proBNP and diabetes mellitus, only LGE+ and Gal3 remained independent predictors for MACEs (*p* = 0.008; *p* = 0.04; Table 4).

Furthermore, gradual logistic regression proportional-hazard models showed a significant incremental predictive ability by adding Gal3 to LGE used alone (from Chi-square = 16.49, *p* < 0.0001 to Chi-square = 21.11, *p* < 0.0001).

**Figure 4:**
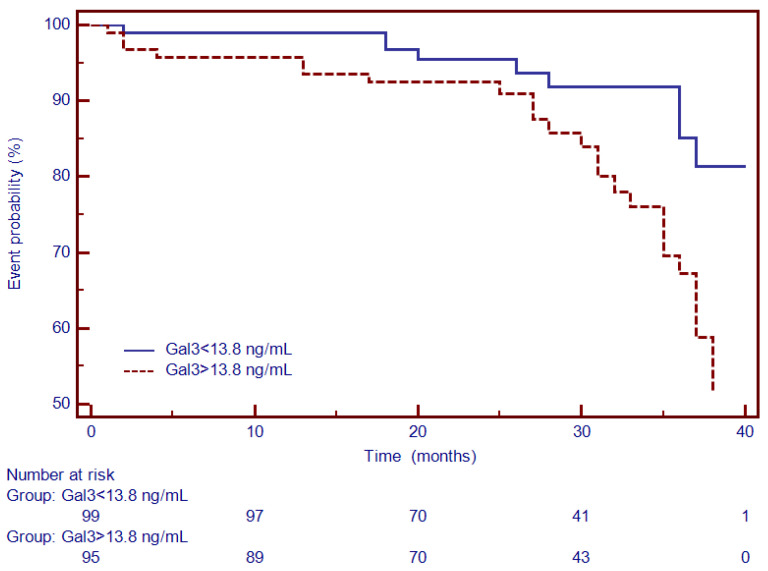
Kaplan–Meier analysis for the ability of Gal-3 to predict cardiovascular outcome. Abbreviations: Gal-3, galectin-3.

**Figure 5:**
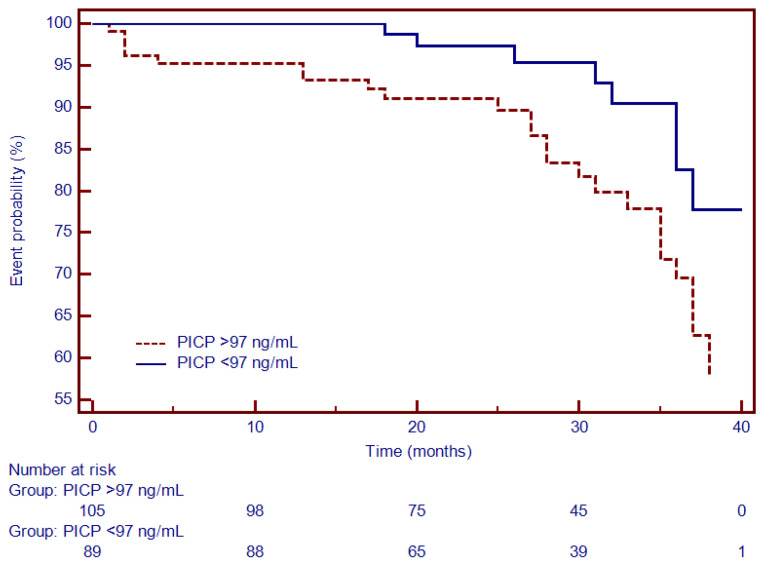
Kaplan–Meier analysis for the ability of PICP to predict cardiovascular outcome. Abbreviations: PICP, procollagen type I carboxy-terminal pro-peptide.

**Figure 6:**
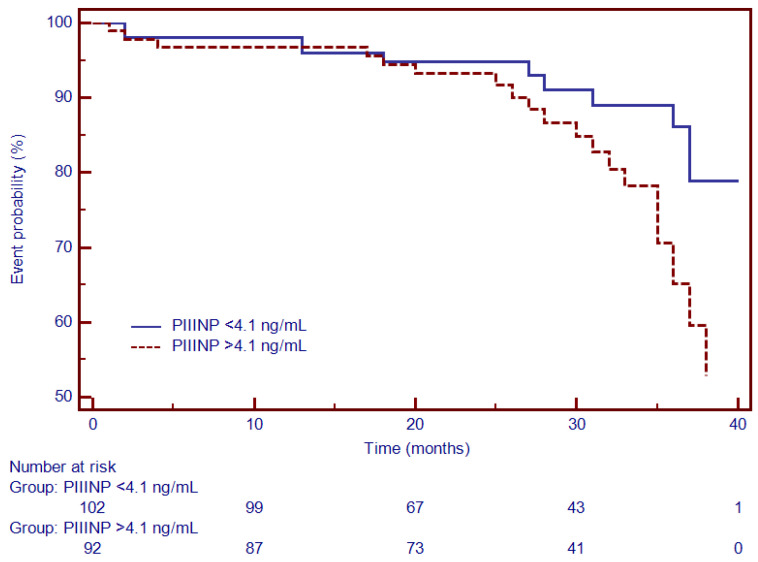
Kaplan–Meier analysis for the ability of PIIINP to predict cardiovascular outcome. Abbreviations: PIIINP, N-terminal pro-peptide of procollagen type III.

**Figure 7:**
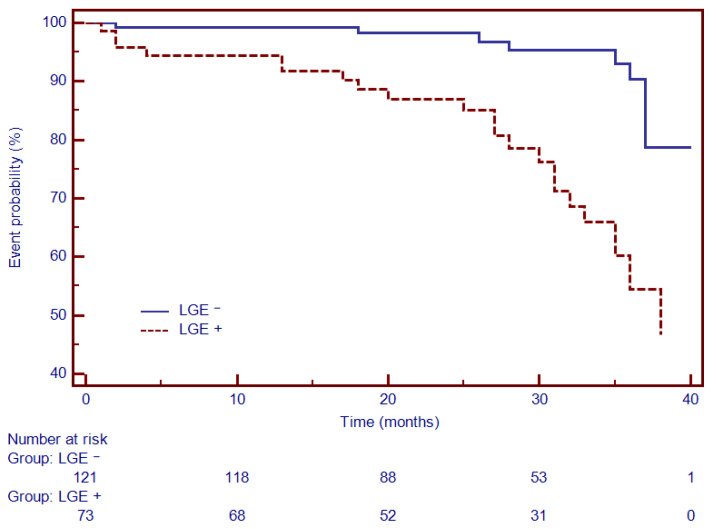
Kaplan–Meier analysis for the ability of LGE to predict cardiovascular outcome. Abbreviations: LGE, late gadolinium enhancement.

Moreover, Kaplan–Meier analysis was performed to test the predictive ability of LGE, Gal3, PICP and PIIINP to predict the composite outcome. Thus, for specific thresholds, circulating collagen turnover biomarkers significantly predicted MACEs: >13.8 ng/mL for Gal3 (HR = 2.66, 95% CI (1.34–5.27), *p* < 0.001; Figure 4), >98 ng/dL for PICP (HR = 1.93, 95% CI (1.17–3.87), *p* < 0.002; Figure 5) and >4.1 ng/dL for PIIINP (HR = 1.42, 95% CI (1.07–2.81), *p* < 0.03; Figure 6), while LGE was also associated with a considerably increased risk of MACEs (HR = 4.06, 95% CI (1.99–8.26), *p* = 0.0001; Figure 7).

**Figure 8:**
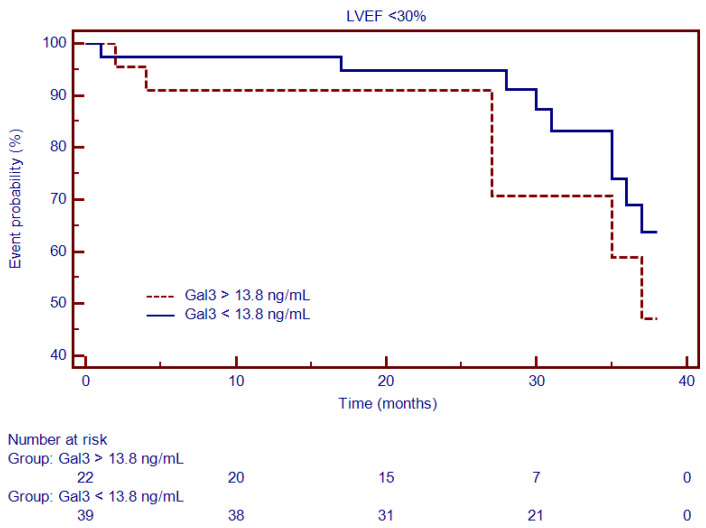
Kaplan–Meier analysis for the ability of Gal-3 to predict cardiovascular outcome in patients with NIDCM and severely decreased LVEF <30%. Abbreviations: Gal-3, galectin-3.

**Figure 9:**
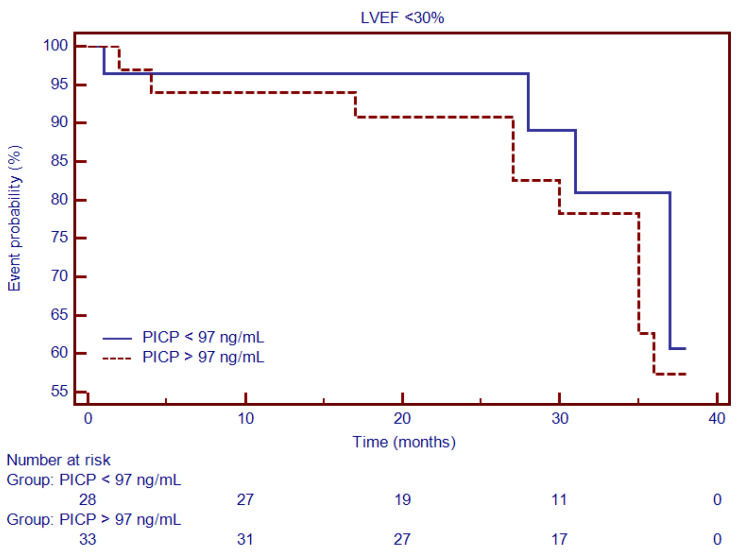
Kaplan–Meier analysis for the ability of PICP to predict cardiovascular outcome in patients with NIDCM and severely decreased LVEF <30%. Abbreviations: PICP, procollagen type I carboxy-terminal pro-peptide.

Moreover, a subgroup analysis that included patients with NIDCM and severely decreased LVEF (<30%) showed that, for similar cut-off values, Gal3 (Figure 8) and PICP (Figure 9) had an even higher predictive ability for outcome: HR = 4.27, 95% CI (2.58–7.06), *p* < 0.0001 and HR = 3.23, 95% CI (1.91–5.46), *p* < 0.0001.

## 4. Discussion

In this study, we evaluated the association of circulating collagen turnover biomarkers with replacement myocardial fibrosis and with severely decreased LV systolic function, both determined by CMR, in patients with NIDCM. The main findings of our research article comprise: (1) sera levels of collagen turnover biomarkers, namely Gal3, PICP and PIIINP, were closely associated with CMR parameters of LV systolic dysfunction, such as LVEDV, LVESV, LVSI, LV-LAS—also being notably correlated with markers of HF severity, namely NYHA class ≥ III, NT-proBNP and CPP levels; (2) Gal3, PICP and PIIINP were directly associated with the mass of replacement myocardial fibrosis, represented as LGE mass and the LGE mass/LV mass ratio; (3) along with LGE, Gal3 and PICP were the most notable independent predictors of cardiovascular outcome; (4) the addition of Gal3, PICP and PIIINP provided an incremental ability to diagnose severely decreased LV systolic function in this category of patients.

Cardiac fibrosis is frequently found in patients with NIDCM and is associated with a more aggressive disease phenotype, being more difficult to treat. At the root of these findings stands accelerated progression of LV dysfunction, congestive HF, and increased risk of sudden cardiac death [1,23]. Previously published studies have shown that in patients with NIDCM, the presence and extent of LGE were independently associated with HF, malignant ventricular tachyarrhythmias, cardiac death and all-cause mortality [1,5,24,25]. Nonetheless, LGE has several limitations in detecting myocardial scarring; thus, by corroborating CMR with sera biomarkers, it might increase diagnostic accuracy [23]. In our current study, we have shown that the combined use of LGE with collagen turnover biomarkers significantly increased prognosis prediction and risk stratification in patients with NIDCM.

Amongst all the markers, Gal3 had the strongest predictive ability, being an independent predictor for outcome, together with LGE—even after the adjustment for standard covariates such as age, gender, LVEF, NYHA class, renal function, and NT-proBNP. Besides this, in our study, we have shown that circulant Gal3 was independently associated with myocardial fibrosis, quantified as LGE by CMR in patients with NIDCM—this being another innovative aspect of our research. Similarly, Vergaro et al. have shown that plasmatic levels of Gal3 are closely associated with LGE in patients with NIDCM [15]. Additionally, a recently published murine study has shown that the suppression of Gal3 has beneficial effects on the regression of NIDCM [26].

Sera PICP was reported to be an important circulant marker of type I collagen turnover, being significantly associated with myocardial fibrosis [27] and with an increased risk of MACEs in patients with NIDCM. Nonetheless, in formerly published studies, the prognostic ability of myocardial fibrosis is rather questionable due to their contradictory results [28,29]. In our study, PICP and PIIINP were closely associated with LGE mass and proved to have a significant ability to predict the occurrence of MACEs; however, in the Cox analysis—after the adjustment for confounders—none of them remained independent predictors for outcome.

Furthermore, we evaluated the profile of these biomarkers in patients with NIDCM and severely decreased LVEF of under 30%. All of them had significantly increased sera levels and were even closely related to HF parameters. Moreover, the stepwise addition of these biomarkers to LGE proved to increase their association with decreased LVEF, beyond that of each parameter used alone. Thus, our study suggests the utility of these sera biomarkers even in the risk stratification of these patients.

Furthermore, the joint use of circulating biomarkers and LGE might become useful in monitoring disease progression and also in identifying patients who would benefit from implantable cardioverter devices or cardiac resynchronization therapy [30,31], but these things are only in their infancy.

Withal, an important issue that needs to be considered is that these biomarkers reflect the systemic metabolism of collagen, and not only in cardiac collagen; thus, this is the reason why these markers could become useful in heart diseases only when they can be combined with cardiovascular imaging parameters [4]. Likewise, further research should focus on exploring if the combined use of CMR with circulant collagen turnover biomarkers might aid in therapeutic monitoring and cardiovascular risk stratification in patients with NIDCM.

### Study Limitations

Firstly, being a single-center study represents a limitation by default. Secondly, T1-maps and extracellular volumes were not assessed in all patients since this technique was not available in our research facility at the beginning of the study. Lastly, the long recruitment period might have affected the sera samples from which the biomarkers were determined.

## 5. Conclusions

In patients with NIDCM, circulating collagen turnover biomarkers—namely Gal3, PICP and PIIINP—were independently associated with myocardial replacement fibrosis determined as LGE by CMR, being useful in the risk stratification of them. These markers were even higher in those with NIDCM and severely decreased LVEF. Moreover, they were useful in prognosis prediction; however, only Gal3 proved to be an independent predictor for cardiovascular outcome.

## Figures and Tables

**Table 1:** Baseline characteristics.

Data	All Patients (n = 194)	LGE− (n = 121)	LGE+ (n = 73)	*p*
Clinical features				
Age, mean (SD), years	48.7 (14.3)	47.9 (14.7)	50.0 (13.6)	NS
Masculine gender, *n* (%)	144 (74.2)	88 (72.7)	56 (76.7)	NS
BMI, Kg/m^2^	27.4 (4.7)	27.2 (4.5)	27.6 (5.1)	NS
HR, mean (SD), bpm	73 (16.0)	70 (14.2)	76 (17.9)	NS
SBP, mean (SD), mmHg	134 (19.1)	135 (18.5)	131 (19.7)	NS
AHT, *n* (%)	102 (52.5)	72 (59.5)	30 (41.0)	<0.05
Diabetes mellitus, *n* (%)	63 (32.5)	43 (35.5)	20 (27.4)	<0.05
Dyslipidemia, *n* (%)	111 (57.2)	70 (57.8)	41 (56.2)	NS
Smokers, *n* (%)	65 (33.5)	41 (33.8)	24 (32.8)	NS
NYHA I/II/III class	30/97/37	20/59/23	10/38/14	<0.05
Medication				
Betablockers, *n* (%)	149 (76.8)	93 (76.8)	56 (76.7)	NS
ACEI or ARB2, *n* (%)	147 (75.7)	92 (76.0)	56 (76.7)	NS
Calcium channel blockers, *n* (%)	32 (16.5)	20 (16.5)	12 (16.4)	NS
Diuretics, *n* (%)	118 (60.8)	73 (60.3)	45 (61.4)	NS
Biomarkers				
NT-proBNP, median (IQR), ng/L	16,900 (8700–39,500)	16,200 (8700–36,200)	17,200 (10,600–39,500)	<0.001
CPP, median (IQR), ng/mL	12.7 (1.8–87)	8.2 (1.8–68.2)	17.1 (4.3–87)	<0.001
PICP, median (IQR), ng/mL	97 (23–347)	74 (23–344)	156 (38–347)	<0.001
PIIINP, median (IQR), ng/mL	4.1 (1.7–8.7)	3.5 (1.7–7.1)	5.1 (2.1–8.7)	<0.001
Gal3, median (IQR), ng/mL	13.8 (2.2–26.6)	9.1 (2.2–23.6)	17.7 (6.1–26.6)	<0.001
eGFR, mean (SD), mL/min/1.73 m^2^	87.1 (21.2)	87.7 (20.4)	86.1 (22.6)	NS
CMR				
LVEDV indexed, median (SD), mL/m^2^	131.1 (34.5)	124.2 (29.7)	142.4 (39.1)	<0.001
LVESV indexed, median (SD), mL/m^2^	86.8 (33.7)	78.1 (28.4)	101.2 (36.9)	<0.001
LVM indexed, median (SD), g/m^2^	86.1 (20.5)	83.3 (19.4)	90.5 (21.6)	<0.01
LVEF, median (SD), %	35.2 (9.6)	38.2 (7.8)	30.3 (9.3)	<0.001
LAV indexed, median (SD), mL/m^2^	55.5 (21.4)	53.1 (20.4)	60.6 (22.2)	<0.05
LV-LAS, median (SD), %	−9.7 (5.3)	−10.5 (5.1)	−8.5 (5.4)	<0.001
LVSI, median (SD)	0.41 (0.13)	0.38 (0.15)	0.44 (0.12)	<0.001
LGE mass, median (IQR), g	-	-	31.2 (1–89)	N/A
LGE mass/LVM, median (IQR), %	-	-	18.4 (0.6–56)	N/A

Abbreviations: ACEI, angiotensin-converting enzyme inhibitors; AHT, arterial hypertension; ARB2, angiotensin II receptor blockers; BMI, body-mass index; CPP, copeptin; eGFR, estimated glomerular filtration rate; Gal3, Galectin-3; HR, heart rate; IQR, interquartile range; LAS, left ventricle long-axis strain; LAV, left atrial volume; LGE, late gadolinium enhancement; LVEDV, left ventricle end-diastolic volume; LVEF, left ventricle ejection fraction; LVESV, left ventricle end-systolic volume; LVM, left ventricle mass; LVSI, left ventricle sphericity index; NYHA, New York Heart Association; PICP, procollagen type I carboxy-terminal pro-peptide; PIIINP, N-terminal pro-peptide of procollagen type III; SBP, systolic blood pressure; SD, standard deviation.

**Table 2:** Comparison between NIDCM patients with severely and non-severely decreased LVEF.

Data	All Patients (*n* = 194)	LVEF 31–45% (*n* = 131)	LVEF < 30% (*n* = 61)	*p*
NT-proBNP, median (IQR), ng/L	16,900 (8700–39,500)	16,200 (8700–36,200)	17,500 (10,500–39,500)	<0.01
CPP, median (IQR), ng/mL	12.7 (1.8–87)	9.5 (1.8–68.2)	17.5 (3.2–87)	<0.001
PICP, median (IQR), ng/mL	97 (23–347)	79 (23–344)	147 (32–347)	<0.001
PIIINP, median (IQR), ng/mL	4.1 (1.7–8.7)	3.9 (1.7–8.7)	4.5 (1.9–8.7)	<0.001
Gal3, median (IQR), ng/mL	13.8 (2.2–26.6)	9.6 (2.2–26.6)	17.7 (3.1–23.6)	<0.001
eGFR, mean (SD), mL/min/1.73 m^2^	87.1 (21.2)	87.7 (20.4)	86.1 (22.6)	NS
LVEDV indexed, median (SD), mL/m^2^	131.1 (34.5)	117.4 (21.6)	160.7 (38.8)	<0.001
LVESV indexed, median (SD), mL/m^2^	86.8 (33.7)	69.9 (15.7)	124.2 (32.3)	<0.001
LVM indexed, median (SD), g/m^2^	86.1 (20.5)	80.9 (17.7)	97.1 (21.9)	<0.01
LAV indexed, median (SD), mL/m^2^	55.5 (21.4)	51.7 (19.5)	63.7 (22.8)	<0.01
LAS, median (SD), %	−9.7 (5.3)	−11.6 (5.1)	−5.7 (2.5)	<0.001
LVSI, median (SD)	0.41 (0.13)	0.37 (0.09)	0.46 (0.13)	<0.001
LGE mass, median (IQR), g	14.2 (0.9–88)	6.4 (0.9–71.1)	31.2 (1–88)	<0.001
LGE mass/LVM, median (IQR), %	8.8 (0.6–64.2)	4.5 (0.6–44.7)	18.4 (16.9–64.2)	N/A

Abbreviations: ACEI, angiotensin-converting enzyme inhibitors; AHT, arterial hypertension; ARB2, angiotensin II receptor blockers; BMI, body-mass index; CPP, copeptin; eGFR, estimated glomerular filtration rate; Gal3, Galectin-3; HR, heart rate; IQR, interquartile range; LAS, left ventricle long-axis strain; LAV, left atrial volume; LGE, late gadolinium enhancement; LVEDV, left ventricle end-diastolic volume; LVEF, left ventricle ejection fraction; LVESV, left ventricle end-systolic volume; LVM, left ventricle mass; LVSI, left ventricle sphericity index; NYHA, New York Heart Association; PICP, procollagen type I carboxy-terminal pro-peptide; PIIINP, N-terminal pro-peptide of procollagen type III; SBP, systolic blood pressure; SD, standard deviation.

**Table 3:** Comparison between patients with NIDCM who reached MACEs and the others.

Data	All Patients (*n* = 194)	MACEs− (*n* = 161)	MACEs+ (*n* = 33)	*p*
Clinical features				
Age, mean (SD), years	48.7 (14.3)	48.5 (13.6)	49.3 (17.7)	NS
Masculine gender, *n* (%)	144 (74.2)	121 (84.0)	23 (26.0)	NS
BMI, Kg/m^2^	27.4 (4.7)	27.6 (4.7)	26.1 (4.4)	NS
HR, mean (SD), bpm	73 (16.0)	72 (15.4)	75 (18.2)	NS
SBP, mean (SD), mmHg	134 (19.1)	135 (19.2)	131 (17.9)	NS
AHT, *n* (%)	102 (52.5)	87 (85.2)	15 (14.8)	<0.001
Diabetes mellitus, *n* (%)	63 (32.5)	52 (82.5)	11 (17.5)	<0.001
Dyslipidemia, *n* (%)	111 (57.2)	91 (81.9)	20 (18.1)	<0.001
Smokers, *n* (%)	65 (33.5)	57 (87.7)	8 (12.3)	<0.001
NYHA I/II/III class	30/97/37	21/91/31	9/6/6	<0.05
Medication				
Betablockers, *n* (%)	149 (76.8)	124 (83.2)	25 (16.8)	<0.001
ACEI or ARB2, *n* (%)	147 (75.7)	125 (85.0)	22 (15.0)	<0.001
Calcium channel blockers, *n* (%)	32 (16.5)	22 (68.7)	8 (31.3)	<0.001
Diuretics, *n* (%)	118 (60.8)	93 (78.8)	25 (21.2)	<0.001
Biomarkers				
NT-proBNP, median (IQR), ng/L	16,900 (8700–39,500)	14,000 (8700–36,600)	19,300 (10,200–39,500)	<0.001
CPP, median (IQR), ng/mL	12.7 (1.8–87)	9.9 (1.8–87)	16.2 (3.1–82.9)	<0.001
PICP, median (IQR), ng/mL	97 (23–347)	92 (23–347)	118 (32–338)	<0.001
PIIINP, median (IQR), ng/mL	4.1 (1.7–8.7)	4.0 (1.7–8.3)	4.5 (2.1–8.7)	<0.01
Gal3, median (IQR), ng/mL	13.8 (2.2–26.6)	11 (2.2–26.6)	17.2 (3.0–24.0)	0.001
eGFR, mean (SD), mL/min/1.73 m^2^	87.1 (21.2)	86.1 (19.7)	89.6 (25.8)	NS
CMR				
LVEDV indexed, median (SD), mL/m^2^	131.1 (34.5)	130.4 (35.0)	134.7 (32.7)	NS
LVESV indexed, median (SD), mL/m^2^	86.8 (33.7)	85.4 (33.9)	93.7 (32.4)	NS
LVM indexed, median (SD), g/m^2^	86.1 (20.5)	85.9 (20.5)	86.4 (20.6)	NS
LVEF, median (SD), %	35.2 (9.6)	35.9 (9.2)	31.7 (9.1)	<0.01
LAV indexed, median (SD), mL/m^2^	55.5 (21.4)	54.2 (21.7)	61.7 (18.4)	NS
LV-LAS, median (SD), %	−9.7 (5.3)	−10.2 (5.5)	−7.8 (3.5)	<0.01
LVSI, median (SD)	0.41 (0.13)	0.38 (0.11)	0.47 (0.13)	<0.001
LGE mass, median (IQR), g	14.3 (0–89)	11.2 (0–86)	29.9 (23–89)	<0.001
LGE mass/LVM, median (IQR), %	8.4 (0–56)	6.6 (0–52.8)	19.4 (1.2–56)	<0.001

Abbreviations: ACEI, angiotensin-converting enzyme inhibitors; AHT, arterial hypertension; ARB2, angiotensin II receptor blockers; BMI, body-mass index; CPP, copeptin; eGFR, estimated glomerular filtration rate; Gal3, Galectin-3; HR, heart rate; IQR, interquartile range; LAS, left ventricle long-axis strain; LAV, left atrial volume; LGE, late gadolinium enhancement; LVEDV, left ventricle end-diastolic volume; LVEF, left ventricle ejection fraction; LVESV, left ventricle end-systolic volume; LVM, left ventricle mass; LVSI, left ventricle sphericity index; MACEs, major adverse cardiovascular events; NYHA, New York Heart Association; PICP, procollagen type I carboxy-terminal pro-peptide; PIIINP, N-terminal pro-peptide of procollagen type III; SBP, systolic blood pressure; SD, standard deviation.

**Table 4:** Univariate and multivariate Cox analysis for MACEs.

Parameters	Univariate Analysis		Multivariate Analysis	
	HR Unadjusted (95% CI)	*p*	HR Adjusted (95% CI)	*p*
LGE	4.06 (1.94–8.52)	0.0001	4.91 (2.06–11.6)	0.008
Gal3	2.67 (1.32–5.28)	0.008	1.11 (1.03–1.19)	0.04
PICP	1.04 (1.01–1.07)	0.001	1.00 (0.98–1.07)	NS
PIIINP	1.09 (1.02–1.11)	0.001	1.00 (0.98–1.03)	NS

Abbreviations: Gal3, galectin-3; LGE, late gadolinium enhancement; PICP, procollagen type I carboxy-terminal pro-peptide; PIIINP, N-terminal pro-peptide of procollagen type III. Multivariate analysis, after adjustment for covariates which comprised age, gender, LVEF, eGFR, BMI, NT-proBNP, and diabetes mellitus.

## Data Availability

Not applicable.
